# Thoracolumbar labour epidural analgesia in a parturient with permanent gluteal silicone‐like biopolymer infiltration

**DOI:** 10.1002/anr3.70060

**Published:** 2026-04-09

**Authors:** T. Foggi Viligiardi, C. Matteoni, U. Bitossi, M. Micaglio

**Affiliations:** ^1^ Department of Anaesthesia and Critical Care Azienda Ospedaliero‐Universitaria Careggi Florence Italy

**Keywords:** anaesthesia, epidural, labour, obstetric, silicones, tomography, computed, ultrasonography

## Abstract

Gluteal augmentation procedures with permanent soft tissue fillers, including liquid silicone and silicone‐like biopolymers, are widely used. These procedures may be undertaken in settings without strict regulatory oversight. Unlike resorbable materials, permanent fillers can migrate along fascial planes and induce chronic inflammatory reactions, fibrosis and granulomatous reactions, potentially altering normal anatomical structures years after injection. Cranial migration into paraspinal soft tissues has been reported, causing concern regarding neuraxial access, although the implications for neuraxial anaesthesia are unclear. The presence of foreign material in the lumbar region is regarded as a relative or absolute contraindication to epidural or spinal techniques, even in the absence of radiological evidence of epidural space involvement. The key learning points in our case lie in the presence of extensive permanent silicone infiltration within the gluteal and lower lumbar soft tissues and in the anatomical assessment and clinical decision‐making informed by radiology investigations. We report a case of a parturient with documented permanent gluteal silicone infiltration who had previously been considered unsuitable for neuraxial labour analgesia. Radiological re‐assessment enabled the identification of a safe thoracolumbar approach to labour epidural analgesia.

## Introduction

The injection of silicone‐like biopolymer substances for aesthetic purposes is an increasingly documented procedure in clinical practice [1, 2, 3]. Unlike resorbable fillers, permanent biopolymers can migrate through soft tissues and may induce fibrosis, sterile granulomatous reactions and distortion of anatomical planes, even many years after the initial implantation [1, 2, 3]. In obstetric anaesthesia, the presence of such materials may be interpreted as an absolute contraindication to neuraxial anaesthesia or analgesia, resulting in the proposal of vaginal birth without neuraxial analgesia or caesarean birth under general anaesthesia [1, 2, 3]. In the absence of anatomical assessment, clinical uncertainty may result in the avoidance of a neuraxial technique, even when epidural space involvement has not been demonstrated.

Neuraxial ultrasound reduces the incidence of technical failure and improves the safety of epidural catheter placement, particularly in patients with difficult anatomy [4]. In complex scenarios, the use of CompuFlo® (Milestone Scientific Inc., Livingston, NJ, USA), a computerised injection pump providing real‐time pressure feedback at the needle tip, may aid objective identification of the epidural space and may help in overcoming the subjectivity of the manual loss‐of‐resistance technique [5]. We report a case of a patient who received effective labour epidural analgesia which was initially deemed contraindicated due to a previous aesthetic procedure.

## Report

A 34‐year‐old primiparous woman with BMI 23 kg.m^‐2^ presented with an unremarkable obstetric history at 39^+2^weeks' gestation. Her medical history included a right robotic partial nephrectomy for renal cell carcinoma, with regular oncological and radiological follow‐up. Following computerised tomography (CT), the presence and extent of biopolymer material within the paraspinal soft tissues had been identified. She had no history of previous neuraxial anaesthesia.

Over 10 years prior to the current pregnancy, the patient had undergone deep bilateral gluteal injections at a private clinic in South America for aesthetic purposes. An estimated 1500 ml of material had been injected. The substance was documented by the medical team who performed the procedure as hyaluronic acid. However, based on clinical evaluation and radiological findings, it was later identified as a permanent, non‐resorbable silicone‐like biopolymer. In subsequent years, the patient developed episodic local pain and tissue induration. During an anaesthetic pre‐assessment earlier in the current pregnancy at another institution, she had been considered unsuitable for epidural labour analgesia based on CT images suggestive of paraspinal biopolymer migration. The management discussed was limited to either labour without neuraxial analgesia or caesarean birth under general anaesthesia. The patient expressed a clear preference for neuraxial labour analgesia and sought a second opinion regarding its feasibility.

**Figure 1:**
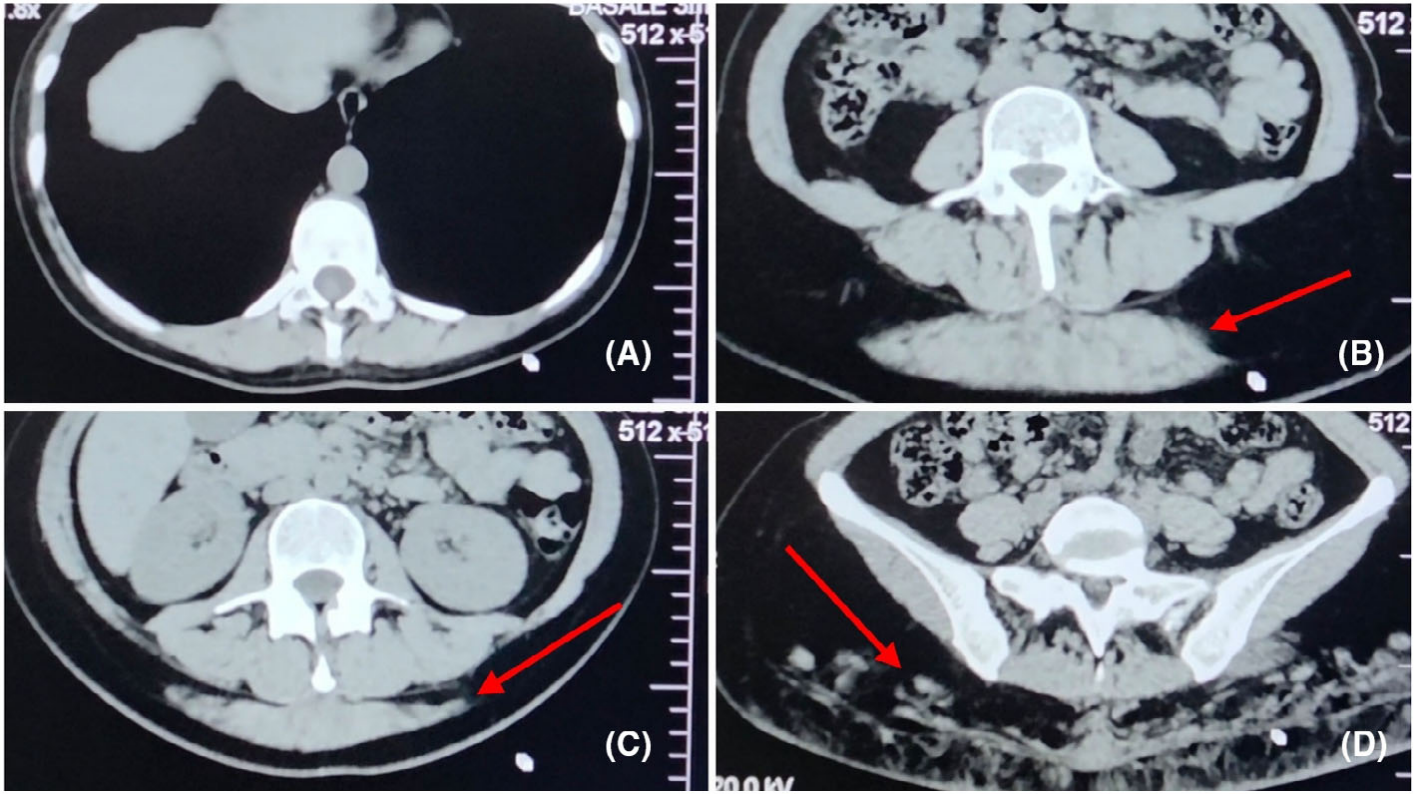
Cranio‐caudal computed tomography sequence demonstrating the anatomical distribution of injected silicone‐like biopolymer material. Red arrows indicate the location of the infiltrating material. (A) L1 vertebral body level: minimal superficial streaks of material within the posterior soft tissues, with preserved posterior vertebral elements and no extension towards the interspinous ligaments, ligamentum flavum or spinal canal. (B) L2 vertebral body level: cranial extent of detectable biopolymer material, appearing as compact deposits confined to superficial posterior paraspinal soft tissues without involvement of posterior ligamentous structures. (C) L3 vertebral body level: dense and well‐defined bilateral accumulation of silicone‐like material within the gluteal and posterior soft tissues, remaining posterior to the vertebral elements and without extension towards the neuraxial compartment. (D) Iliac crest level: caudal distribution showing a more heterogeneous and dispersed pattern within subcutaneous and gluteal planes, while deep paraspinal and central neuraxial structures remain preserved.

At our institution, a detailed review of the most recent CT images was performed by the radiology and anaesthesia teams (Fig. 1).

Hyperdense, well‐demarcated material compatible with the injected permanent silicone‐like biopolymer was identified bilaterally within the gluteal soft tissues. Cranially, the infiltrated material extended to the L2 vertebral body. At the L1 vertebral level, only minimal superficial streaks were observed within the paraspinal muscles, without extension towards the interspinous ligaments or ligamentum flavum.

No evidence of epidural space involvement was detected at any level. The posterior elements, including the spinous processes, interspinous ligaments and ligamentum flavum–dura mater complex, appeared preserved. The epidural space maintained normal radiological characteristics. Selection of the T12–L1 interspace was based on imaging findings demonstrating preserved posterior structures and absence of silicone infiltration at this level, while maintaining an appropriate safety margin relative to the level of the conus medullaris.

Neuraxial ultrasound examination demonstrated a suitable thoracolumbar window at the T12–L1 level, with usual anatomical structures and an estimated epidural depth of 4 cm. Based on these findings, neuraxial labour analgesia using a thoracolumbar paramedian approach was proposed. The patient was counselled regarding the specific uncertainties related to altered soft tissue anatomy, including the potential risk of technical difficulty, inadequate block or unpredictable spread of local anaesthetic. Alternative options, including systemic analgesia and caesarean birth under general anaesthesia in case of failure, were discussed. The patient expressed a clear preference for attempting neuraxial labour analgesia.

The procedure was performed in the sitting position by an experienced consultant anaesthetist who also performed the neuraxial ultrasound assessment. An 18G Tuohy needle was introduced into the T12–L1 interspace via paramedian approach to avoid superficial tissue planes where silicone infiltration was more prominent on imaging, thereby reducing interaction with altered soft tissues during needle advancement. The epidural space was identified using a computerised pressure‐sensing device (CompuFlo®), which provided continuous real‐time monitoring of injection pressure at the needle tip. Progressive increase in tissue resistance was observed during needle advancement, followed by a sustained and characteristic pressure drop at approximately 4 cm from the skin, consistent with identification of the epidural space. After confirming epidural placement, the Tuohy needle was rotated by approximately 180° in order to orient the bevel caudally. This manoeuvre allowed intentional advancement of the epidural catheter in a cranio‐caudal direction, with the aim of directing catheter progression towards the lumbar segments. The epidural catheter was then advanced 4 cm into the epidural space and secured. No complications were noted.

Correct functional positioning of the epidural catheter was confirmed following administration of an initial dose of 20 ml levobupivacaine 0.0625%, which was used as a test dose according to our standard institutional protocol. Catheter position was assessed through incremental dosing and clinical response. Pin‐prick testing demonstrated bilateral sensory block between T10 and L2, consistent with effective analgesia for the first stage of labour. During labour, analgesia was maintained with epidural boluses of 10 ml levobupivacaine 0.0625% combined with sufentanil 0.5 μg.ml^−1^, with a lockout interval of 60 minutes.

The patient remained haemodynamically stable with effective labour analgesia maintained. Following spontaneous vaginal birth, the epidural catheter was removed without complications. The patient was observed on the ward for 36 hours in accordance with standard postpartum care. No neurological deficits or complications were observed, and she was discharged in good clinical condition.

## Discussion

The presence of permanent silicone or silicone‐like biopolymers in the lumbar and paraspinal soft tissues is an increasingly encountered clinical scenario following aesthetic procedures [1, 2, 3]. Migration of injected materials along fascial planes may modify anatomical landmarks relevant to neuraxial procedures. Such alterations may generate uncertainty regarding the safety of epidural access, and in clinical practice, the presence of foreign material may be considered a contraindication to neuraxial techniques. However, available evidence indicates that feasibility depends primarily on the anatomical relationship between the infiltrated material and neuraxial structures rather than on its presence alone [1, 2, 3]. In our case, detailed radiological re‐assessment demonstrated preservation of the posterior ligamentous complex and absence of epidural space involvement, allowing procedural planning to be guided by objective radiological findings.

Computed tomogrpahy provided key information for decision‐making; however, pre‐procedural neuraxial ultrasound also provided point‐of‐care confirmation immediately before needle insertion. Although ultrasound guidance in neuraxial anaesthesia is well established, its routine integration into daily clinical practice remains variable [4, 6]. In this case, ultrasound examination enabled direct visualisation of superficial hyperechoic tissue planes corresponding to silicone deposition and facilitated localisation of the first interspace free from significant infiltration, findings that were consistent with those previously identified on computed tomography. The combination of pre‐existing radiological assessment with real‐time ultrasound evaluation allowed translation of static anatomical information into practical procedural guidance at the bedside.

On the basis of these findings, a thoracolumbar epidural was performed to avoid tissue planes more extensively affected by silicone deposition while maintaining effective labour analgesia. Placement at the T12–L1 interspace was supported by imaging demonstrating minimal cranial extension of the material and intact posterior elements at this level. In obstetric patients, thoracolumbar epidural insertion is physiologically appropriate, as epidural spread typically occurs in a cranial‐caudal direction and provides reliable coverage of the T10–L2 dermatomes during the first stage of labour [7]. However, it should be acknowledged that thoracolumbar epidural placement may be associated with sacral sparing and therefore requires careful clinical assessment to ensure adequate analgesia during the later stages of labour. Selection of a paramedian approach further facilitated access by allowing us to bypass superficial tissue planes more extensively affected by silicone infiltration. Catheter advancement in a caudal direction was performed to optimise drug delivery towards lumbar segments while avoiding traversal of altered superficial planes.

In anatomically modified tissues, tactile feedback during epidural placement may be less predictable, as altered soft tissue characteristics can generate atypical resistance patterns. The use of a computerised pressure‐sensing system provides objective real‐time feedback at the needle tip, which may assist in identifying the epidural space in such contexts, although it does not eliminate the need for careful clinical judgement and does not inherently confer superiority over conventional techniques [5]. This approach is part of our routine clinical practice in cases where anatomical challenges are anticipated. Functional assessment following incremental administration of local anaesthetic demonstrated symmetrical sensory blockade and effective clinical analgesia, supporting correct catheter positioning.

This case illustrates that permanent lumbar silicone infiltration does not necessarily preclude neuraxial labour analgesia when careful imaging review, point‐of‐care ultrasound assessment, anatomical reasoning and appropriate technical modifications are applied. Individualised evaluation allowed safe provision of neuraxial analgesia in a clinical context which might otherwise have been considered unsuitable, emphasising the importance of adapting established neuraxial principles to increasingly complex anatomical presentations encountered in contemporary obstetric anaesthesia practice and promoting patient choice in options for effective labour analgesia.

## Conclusions

Permanent gluteal silicone infiltration should not be considered an absolute contraindication to neuraxial labour analgesia when imaging confirms preservation of neuraxial structures. Careful anatomical assessment combined with appropriate technical adaptation can facilitate safe epidural placement even in the presence of altered soft tissues. This case highlights the importance of individualised evaluation and integration of imaging modalities in contemporary obstetric anaesthesia practice.
